# The relationship between remnant cholesterol and young-onset myocardial infarction in patients with type 2 diabetes: a retrospective study

**DOI:** 10.3389/fphar.2025.1512662

**Published:** 2025-03-17

**Authors:** Yajie Gao, Tianjiao Lei, Peizhu Dang, Yongxin Li

**Affiliations:** ^1^ Department of Cardiovascular Medicine, The First Affiliated Hospital of Xi’an Jiaotong University, Xi’an, China; ^2^ Department of Cardiovascular Surgery, The First Affiliated Hospital of Xi’an Jiaotong University, Xi’an, China

**Keywords:** remnant cholesterol, low-destiny-lipoproteins, young-onset myocardial infarction, type 2 diabetes, biomarker

## Abstract

**Background:**

Remnant cholesterol (RC) has emerged as a novel therapeutic target beyond low-destiny-lipoproteins cholesterol (LDL-c). While elevated RC levels are strongly associated with cardiovascular disease risk in the general population, their specific role in young-onset acute myocardial infarction (AMI) among patients with type 2 diabetes mellitus (T2DM) remains insufficiently explored and warrants further investigation.

**Methods:**

This retrospective study included AMI patients with T2DM admitted to the First Affiliated Hospital of Xi’an Jiaotong University from 2018 to 2022. Patients were stratified into tertiles according to RC levels and compared using thresholds derived the commanded values from the PREDIMED cohort study. The primary outcome was young-onset AMI. Group differences were analyzed using the chi-square test and the Kruskal–Wallis H test, while Spearman correlation analyses assessed relationships between variables. Univariate and multivariate logistic regression analyses were employed to evaluate the association between RC and young-onset AMI.

**Results:**

Among the 2,514 participants (mean age 61.58 ± 11.15 years), 802 (31.9%) had young-onset AMI. The increase of young-onset AMI increased significantly with rising RC levels (27.0% vs 29.7% vs 39.1%, P < 0.001). RC showed significant positive correlation with total cholesterol (TC, r = 0.497, *P* < 0.001), triglycerides (TG, r = 0.411, *P* < 0.001), and LDL-c (r = 0.166, *P* < 0.001). RC was independently associated with a higher risk of young-onset AMI (OR: 1.579; 95% CI: 1.354–1.842; *P* < 0.001), even after adjusting for other traditional risk factors of cardiovascular disease (OR: 1.415; 95% CI 1.189–1.684; *P* < 0.001). Notably, RC levels remained strongly linked to young-onset AMI regardless of whether LDL-c levels were within the desired range.

**Conclusion:**

RC is a significant and independent risk factor for young-onset AMI in T2DM patients, irrespective of LDL-c level. These findings underscore the importance of monitoring and managing RC levels in clinical practice to mitigate cardiovascular risk in this population.

## Introduction

Type 2 diabetes mellitus (T2DM) is a major public health challenge worldwide. The latest statistics shows that there were an estimated 529 million people living with diabetes in 2021, a number will more than double to about 1.31 billion by 2050. Diabetes is particularly prevalent in people 65 and older, but it has also become younger in recent years (Ong et al., 2023). Patients with T2DM have a risk of mortality and cardiovascular events that is 2–4 times greater than that observed in the general population (Rawshani et al., 2018). Atherosclerotic cardiovascular disease (ASCVD) is the leading cause of mortality in adults with T2DM, with acute myocardial infarction (AMI) as one of its most serious consequences (ElSayed et al., 2023). Changes in lifestyle and dietary habits are leading to a younger incidence of ASCVD (including AMI), dramatically worsening both the societal healthcare burden and individual quality of life (Gulati et al., 2020; Mozaffarian, 2016).

Previous studies have demonstrated that dyslipidemia is the key pathological mechanism of AMI (Libby, 2013; Libby, 2021; Kobiyama and Ley, 2018). Low-density lipoprotein cholesterol (LDL-c) serves as a central pathogenic factor, and has become the point of both primary and secondary prevention strategies for cardiovascular diseases (Du et al., 2023). Despite advancements in lipid-lowering therapies, only a limited number of patients achieve optimal control of risk factors in clinical practice, leading to an ongoing increase in residual ASCVD risk, particularly among individuals with diabetes (Sandesara et al., 2019; Xiao et al., 2016; Joshi et al., 2015). In addition to traditional lipid markers, such as total cholesterol (TC), triglyceride (TG), LDL-c, and high-density lipoprotein cholesterol (HDL-c), recent studies have found that Remnant cholesterol (RC), a non-traditional lipid marker, can offer valuable insights into cardiovascular disease risk assessment. RC refers to the cholesterol derived from the degradation of very low-density lipoprotein (VLDL) and intermediate-density lipoprotein (IDL) in the fasting state, as well as the cholesterol in the residue of chylomicrons in the postprandial state. It has been confirmed in lots of studies that remnant cholesterol can exacerbate the formation and progression of atherosclerotic plaques (Salinas and Chapman, 2020; Heo and Jo, 2023; Hoogeveen and Ballantyne, 2021). Several large-scale Mendelian randomizations found a robust causal link between elevated RC levels and increased risk of coronary artery disease and myocardial infarction. RC levels were associated with a 43.12% higher risk of coronary artery disease, and RC was associated with a 2.8-fold higher risk of heart disease. A meta-analysis involving 30,605 patients with coronary heart disease demonstrated that those with elevated RC concentrations had a 54% increased risk of composite endpoint events and a 70% higher risk of MACEs. This highlights the prognostic value of RC in predicting adverse outcomes in patients already diagnosed with heart disease (Huh et al., 2022; Castañer et al., 2020; Wadström et al., 2022). In addition to this, elevated residual cholesterol is associated not only with cardiovascular disease, but also with other cardiometabolic diseases such as T2DM(19). Therefore, RC, as a novel lipid biomarker, is of great significance for the assessment of cardiovascular diseases.

To our knowledge, no previous research has examined the association between RC and young-onset AMI in individuals with T2DM. The study intends to retrospectively enroll patients with T2DM and myocardial infarction admitted to the First Affiliated Hospital of Xi’an Jiaotong University, aiming to clarify the independent risk factor of RC in predicting young-onset AMI in T2DM patients and provide scientific evidence for clinical risk assessment and intervention.

## Methods

### Study design and participants

**FIGURE 1:**
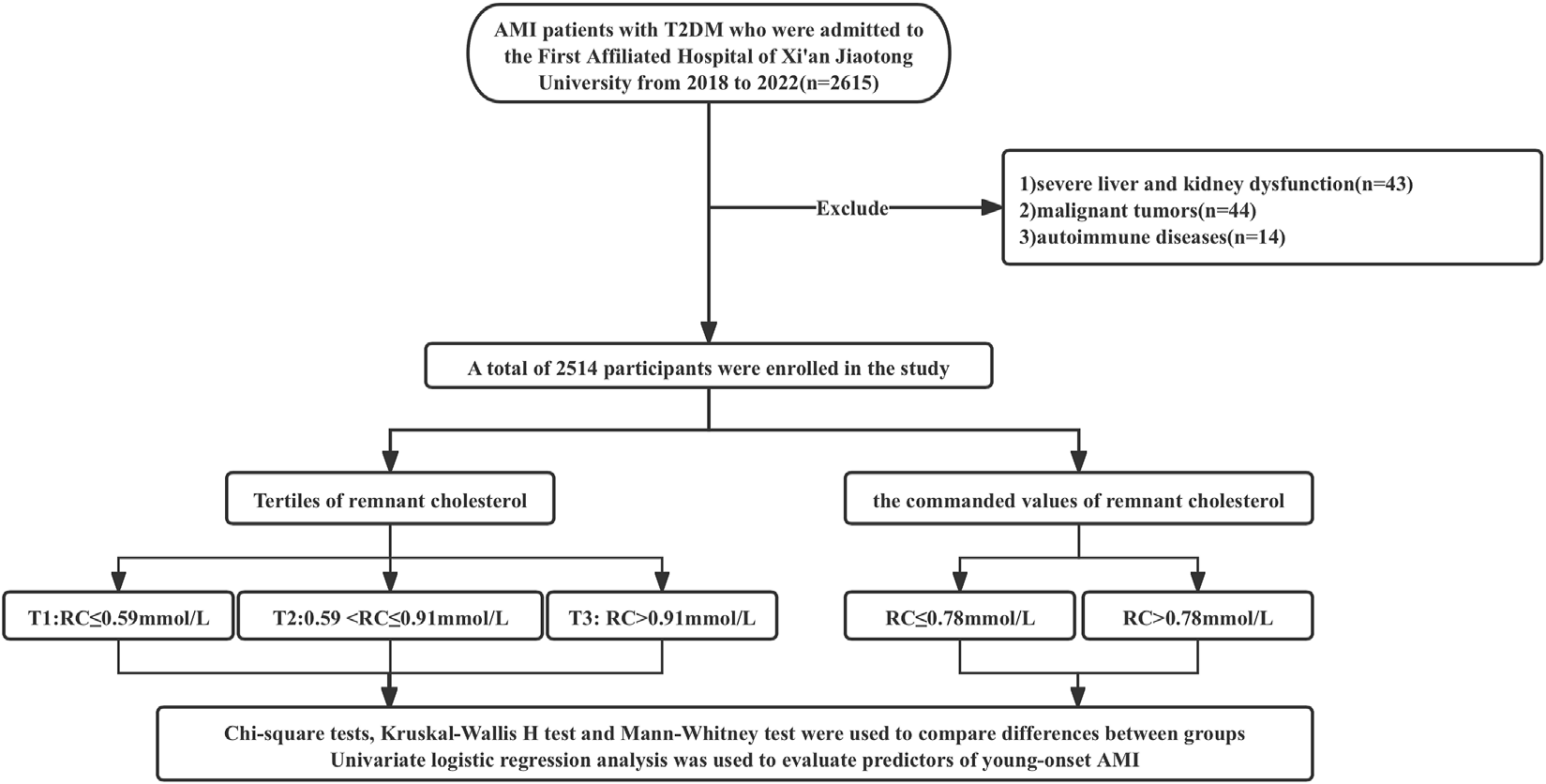
Flow chart of the patient selection and study design.

We performed a retrospective analysis of patients with T2DM and AMI admitted to the First Affiliated Hospital of Xi’an Jiaotong University from 2018 to 2022. The diagnosis of AMI adhered to the Fourth Universal Definition of Myocardial Infarction (2018) (Thygesen et al., 2018), characterized by acute myocardial injury accompanied by clinical evidence of acute myocardial ischemia, and the detection of a rise and/or fall in cardiac troponin levels, with at least one value exceeding the 99th percentile upper reference limit, along with at least one of the following criteria: 1) Symptoms of myocardial ischemia; 2) New ischemic electrocardiogram changes; 3) Development of pathological Q waves; 4) Imaging evidence of new loss of viable myocardium or new regional wall motion abnormality. T2DM was defined as Hba1c ≥ 6.5% (48 mmol/mol) or fasting plasma glucose (FPG)≥7.0 mmol/L (126 mg/dL) (Cosentino et al., 2020). The main exclusion criteria were as follows: 1) severe liver and kidney dysfunction, 2) malignant tumors, and 3) patients with autoimmune diseases. Figure 1 describes the patient selection and study design. This study was approved by the Human Research and Ethics Committee of the First Affiliated Hospital of Xi’an Jiaotong University.

### Data collection and definitions

We collected baseline and clinical characteristics, including demographic data, past medical history, vital signs at admission, laboratory index, echocardiography results, and angiography variables) from the medical record system. Body mass index (BMI) was calculated as weight divided by the square of height in kg/m^2^. The estimated glomerular filtration rate (eGFR) was calculated using the MDRD study equation (Levey et al., 1999; Levey et al., 2006). Venous blood samples were collected at room temperature within 24 h of admission for each subject. Echocardiography was performed to record left ventricular ejection fraction (LVEF), which was used to evaluate the left ventricular systolic function. Young-onset AMI was defined as the manifestation in men below the age of 55 years and women below the age of 65 years (Expert Panel on Detection et al., 2001; Cole and Sperling, 2004). Remnant cholesterol was calculated using the following formula: RC = TC- (HDL-c) - (LDL-c) (units, mmol/L) (Heo and Jo, 2023; Wadström et al., 2023; Solnica et al., 2024; Zhao et al., 2024). This method offers a rough estimation of the RC (Castañer et al., 2020; Nordestgaard et al., 2016). A notable advantage of this approach is its cost-effectiveness and convenience for clinical applications.

### Statistical analysis

In our primary analyses, we divided the participants into three groups according to the tertiles of RC (T1:RC ≤ 0.59 mmol/L, T2: 0.59 <RC ≤ 0.91 mmol/L, and T3: RC > 0.91 mmol/L), as well as two groups according to the PREDIMED cohort study (low: RC ≤ 0.78 mmol/L, high: RC > 0.78 mmol/L) (Castañer et al., 2020). Categorical variables were reported as counts (percentages) and compared using the Chi-square test. Continuous variables with normal distribution were expressed as mean ± SD and compared using variance analysis and a one-way ANOVA test. Continuous variables without normal distribution were expressed as median (interquartile range) and compared using the Kruskal–Wallis H test and Mann-Whitney test. For the selection of logistic regression variables, we determined them on the basis of clinical relevance and statistical significance. Univariate logistic regression analysis was used to evaluate predictors of young-onset AMI. Multivariate logistic regression analysis was performed to identify the independent predictors of young-onset AMI, which was adjusted for gender, systolic blood pressure (SBP), diastolic blood pressure (DBP), body mass index (BMI), myocardial infarction history, glycosylated hemoglobin (HAb1c), creatine kinase isoenzyme (CKMB), and eGFR. The results are expressed as odds ratios (OR) with the corresponding 95% confidence intervals (CI). All tests were 2-tailed, and statistical significance was set at P < 0.05. The missing values were imputed using the multiple imputation methods. All statistical analyses were performed using the SPSS 26.0(IBM Corporation).

## Results

### Characteristic of study participants

A total of 2,514 participants were enrolled in the study, including 1900 men with the average age of 59.87 ± 11.11, and 614 women with average age of 66.88 ± 9.52. There were 802 (31.9%) patients with young-onset AMI. We divided the study participants into three groups according to the tertiles of RC levels. Baseline and clinical characteristics are summarized in Table 1. Statistically significant variations were seen in age, SBP, DBP, and BMI. Patients with elevated RC levels exhibited increased TC, TG, and LDL-c, alongside decreased HDL (*P* < 0.05). With the increase of RC, the number of young-onset AMI also increased significantly. Supplementary Table S1 presents the comparison of various biochemical indicators and coronary artery lesions. No statistical difference was seen between the groups regarding complications and diseased vessels.

**TABLE 1:** Baseline characteristics of study participants.

Variables	T1 (n = 838)^a^	T2 (n = 843)^a^	T3 (n = 833)^a^	*P*	RC ≤ 0.78 mmol/L (n = 1,470)^b^	RC > 0.78 mmol/L (n = 1,044)^b^	*P*
Men, n (%)	653 (77.9)	636 (75.4)	611 (73.3)^c^ ^,^ ^d^	0.093	1,133 (77.0)	767 (73.4)	0.038
Age (years)	63 (56, 70)	63 (54, 69)	60 (52, 69)^c^ ^,^ ^d^	<0.001	63 (55, 70)	61 (52, 69)	<0.001
SBP (mmHg)	128 (113, 143)	126 (111, 143)	130 (113, 146)^d^	0.037	127 (112, 143)	129 (113, 145)	0.034
DBP (mmHg)	77 (69, 87)	77 (69, 87)	80 (70, 91)^c^ ^,^ ^d^	<0.001	77 (69, 87)	80 (69, 90)	<0.001
BMI (kg/m^2^)	24.46 (22.48, 26.45)	24.69 (22.86, 26.45)	25.18 (23.15, 27.29)^c^ ^,^ ^d^	0.003	24.49 (22.6, 26.47)	25.04 (23.05, 27.04)	0.003
NSTEMI, n (%)	416 (49.6)	403 (47.8)	353 (42.4)^c^ ^,^ ^d^	0.008	718 (48.8)	454 (43.5)	0.008
STEMI, n (%)	422 (50.4)	440 (52.2)	480 (57.6)^c^ ^,^ ^d^		752 (51.2)	590 (56.5)	
Killip, n (%)				<0.001			0.001
I	598 (71.9)	599 (71.9)	670 (81.0)		1,050 (72.1)	817 (78.9)	
II	160 (19.2)	161 (19.3)	100 (12.1)		282 (19.4)	138 (13.4)	
III	42 (5.0)	42 (5.0)	31 (3.7)		69 (4.7)	46 (4.4)	
IV	32 (3.8)	31 (3.7)	26 (3.1)		56 (3.8)	33 (3.2)	
Young-onset AMI, n (%)	226 (27.0)	250 (29.7)	326 (39.1)^c^ ^,^ ^d^	<0.001	409 (27.8)	393 (37.6)	<0.001
HA1bc (%)	7.6 (6.7, 8.9)	7.9 (7.0, 9.2)	8.0 (7.0, 9.5)^c^ ^,^ ^d^	<0.001	7.7 (6.8, 9.1)	8 (7, 9.4)	<0.001
GLU (mmol/L)	9.56 (7.04, 12.93)	10.31 (7.46, 13.65)	10.57 (7.76, 14.2)^c^	<0.001	9.78 (7.15, 13.12)	10.64 (7.82, 14.19)	<0.001
LDL-c (mmol/L)	2.08 (1.55, 2.74)	2.24 (1.72, 2.87)	2.45 (1.94, 3.10)^c^ ^,^ ^d^	<0.001	2.14 (1.6, 2.77)	2.45 (1.93, 3.1)	<0.001
HDL-c (mmol/L)	0.91 (0.77, 1.05)	0.87 (0.75, 1.02)	0.87 (0.73, 1.01)^c^	<0.001	0.89 (0.77, 1.04)	0.87 (0.73, 1.01)	<0.001
TG (mmol/L)	1.16 (0.85, 1.49)	1.47 (1.08, 1.98)	1.87 (1.25, 2.87)^c^ ^,^ ^d^	<0.001	1.25 (0.94, 1.68)	1.81 (1.24, 2.71)	<0.001
TC (mmol/L)	3.43 (2.82, 4.16)	3.84 (3.3, 4.59)	4.72 (4.06, 5.52)^c^ ^,^ ^d^	<0.001	3.61 (3.01, 4.32)	4.62 (3.9, 5.4)	<0.001
Lp (a) (mmol/L)	222 (118, 392)	206 (115, 392)	202 (106.3, 388.3)	0.956	212 (115, 393)	202 (112, 382.8)	0.815
ApoE (mmol/L)	31.15 (24.5, 39)	34.3 (27.4, 42.5)	38.9 (30.2, 51.88)^c^ ^,^ ^d^	<0.001	32.1 (25.5, 40.45)	38.4 (30.1, 50.4)	<0.001
ApoB (mmol/L)	0.73 (0.59, 0.89)	0.81 (0.68, 0.97)	0.88 (0.73, 1.04)^c^ ^,^ ^d^	<0.001	0.75 (0.61, 0.91)	0.87 (0.73, 1.04)	<0.001
ApoA (mmol/L)	1.03 (0.91, 1.16)	1.02 (0.91, 1.16)	1.04 (0.93, 1.17)^d^	0.391	1.02 (0.91, 1.16)	1.04 (0.92, 1.17)	0.424
RC (mmol/L)	0.47 (0.37, 0.53)	0.72 (0.65, 0.81)	1.24 (1.05, 1.58)^c^ ^,^ ^d^	<0.001	0.56 (0.45, 0.67)	1.13 (0.95, 1.47)	<0.001

Continuous variables are shown as M±SD, or quartiles, and classification variables are shown as percentages.

^a^ The subjects were divided into three groups according to remnant cholesterol tertiles.

^b^ The subjects were divided into two groups according to the PREDIMED, cohort study. The chi-square test and Kruskal–Wallis H test were used for statistical analysis.

^c^ Compared with T1 group, there was a statistical difference.

^d^ Compared with T2 group, there was a statistical difference.

SBP, systolic arterial pressure; DBP, diastolic arterial pressure; BMI, body mass index; STEMI, ST-elevation myocardial infarction; NSTEMI, Non-ST-elevation myocardial infarction; HA1bc, Glycated hemoglobin; TC, total cholesterol; LDL-c, Low-density lipoprotein cholesterol; HDL-c, High-density lipoprotein cholesterol; TG, Triglyceride. ApoE, apolipoprotein E; ApoB, apolipoprotein; ApoA, apolipoprotein A; RC, remnant cholesterol.

### Correlations of RC with variables

A significant positive correlation was found between the RC and the level of TC (r = 0.497, *P* < 0.001), TG (r = 0.411, *P* < 0.001), LDL-c (r = 0.166, *P* < 0.001), GLU (r = 0.118, *P* < 0.001), and BMI (r = 0.066, P = 0.001). The RC level was significantly negatively correlated with HDL (r = −0.102, *P* < 0.001). However, an association between the RC and level of hs-TnT was not significant (Table 2).

**TABLE 2:** Correlation between RC and other traditional risk factors.

Variables	Correlation coefficients	*P*
TC	0.497	<0.001
TG	0.411	<0.001
LDL-c	0.166	<0.001
HDL-c	−0.102	<0.001
Lp (a)	−0.046	0.022
NT-proBNP	−0.114	<0.001
Hs-TnT	0.008	0.702
GLU	0.118	<0.001
BMI	0.066	0.001

Spearman correlation analysis was used to explore the relationship between RC, and variables.

TC, total cholesterol; TG, triglyceride; LDL-c, Low-density lipoprotein; HDL, High-density lipoprotein; Lp (a), lipoprotein(a); NT-proBNP, N-terminal pro-B, type natriuretic peptide; Hs-TnT, Hypersensitive troponin T; GLU, glucose; BMI, body mass index.

### Associations between variables and young-onset AMI

Univariate analysis results indicated that age, gender, SBP, DBP, BMI, STEMI, MI history, HAb1c, CKMB, and eGFR were associated with young-onset AMI (Table 3). RC was identified as a significant risk factor for young-onset AMI (OR:1.579; 95% CI 1.354, 1.842; *P* < 0.001). Multivariable logistic regression indicated that RC was an independent predictor of young-onset AMI, even after controlling for age, gender, SBP, DBP, BMI, MI history, and other conventional cardiovascular disease risk variables (OR:1.415; 95% CI 1.189, 1.684; *P* < 0.001, Table 4). At the same time, we observed that the risk of young-onset AMI in the T3 group was significantly higher than that in the T1 group (OR:1.576; 95% CI 1.124, 2.211; *P* < 0.05), while there was no statistical difference in the T2 group (OR:1.216; 95% CI 0.896, 1.773; *P* > 0.05). The statistical results of the other classification methods are also similar. We also test the collinearity problem in the model by variance inflation factor and correlation matrix, and the results show that the variables in our model do not exist collinearity and have good stability.

**TABLE 3:** Associations between variables and young-onset AMI.

Variables	OR	95% CI	*P*
Age	0.619	0.591, 0.647	<0.001
Men	1.71	1.387, 2.107	<0.001
SBP	0.997	0.993, 1.001	0.095
DBP	1.03	1.023, 1.036	<0.001
BMI	1.148	1.105, 1.193	<0.001
STEMI	1.437	1.213, 1.703	<0.001
MI history	2.026	1.420, 2.889	<0.001
Hypertension	0.626	0.527, 0.743	<0.001
HAb1c	1.076	1.026, 1.129	0.003
GLU	1.012	0.995, 1.030	0.163
CKMB	1.001	1.000, 1.002	0.003
GFR	1.017	1.014, 1.019	<0.001
BUN	0.891	0.862, 0.922	<0.001
RC	1.579	1.354, 1.842	<0.001

OR, odds ratios; CI, confidence interval; SBP, systolic arterial pressure; DBP, diastolic arterial pressure; BMI, body mass index; STEMI, ST-elevation myocardial infarction; HA1bc, Glycated hemoglobin; GLU, glucose; CKMB, creatine kinase isoenzyme; GFR, glomerular filtration rate; BUN, blood urea nitrogen; RC, remnant cholesterol.

**TABLE 4:** Associations between RC and young-onset AMI.

Variable	Crude		Model 1		Model 2	
	ExpB (95% CI)	*P*	ExpB (95% CI)	*P*	ExpB (95% CI)	*P*
RC	1.579 (1.354, 1.842)	**<0.001**	1.431 (1.089, 1.881)	**0.01**	1.415 (1.189, 1.684)	**<0.001**
RC						
T1	reference		reference		reference	
T2	1.142 (0.923, 1.412)	0.222	1.193 (0.866, 1.643)	0.28	1.261 (0.896, 1.773)	0.215
T3	1.741 (1.416, 2.141)	**<0.001**	1.592 (1.160, 2.183)	**0.004**	1.576 (1.124, 2.211)	**0.008**
RC						
≤0.78 mmol/L	reference		reference		reference	
RC						
>0.78 mmol/L	1.566 (1.322, 1.855)	**<0.001**	1.465 (1.128, 1.903)	**0.004**	1.390 (1.051.1.838)	**0.021**

OR, odds ratios; CI, confidence interval. P < 0.05 was statistically significant.

Model 1: adjusted for gender, SBP, DBP, BMI, MI, history.

Model 2: Model 1, additionally adjusted for HAblc, CKMB, eGFR.

RC, remnant cholesterol; AMI, acute myocardial infarction.

### Subgroup analysis of RC and young-onset AMI

**FIGURE 2:**
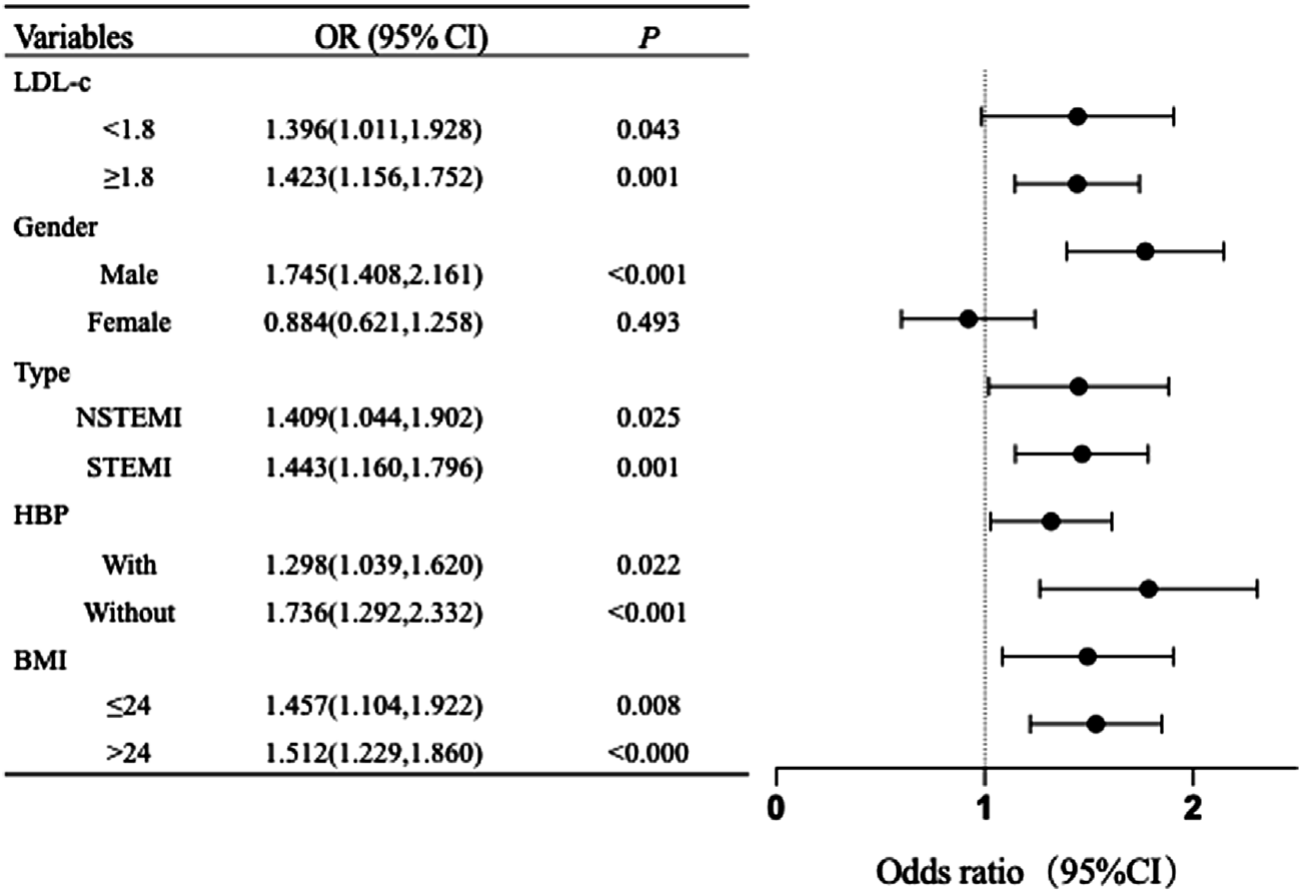
Subgroups analysis of RC and young-onset AMI The model was adjusted for gender, SBP, DBP, BMI, MI history, HAblc, CKMB, and eGFR. *P* < 0.05 indicated that the difference was statistically significant. In each layer, all models are not adjusted for the variables themselves. OR, odds ratios; CI, confidence interval; LDL, Low-density lipoprotein; STEMI, ST-elevation myocardial infarction; NSTEMI, Non-ST-elevation myocardial infarction; HBP, hypertension; BMI, body mass index.

We investigated the correlation between RC and young-onset AMI by categorizing participants into specific groups according to gender, BMI, hypertension, LDL-c levels, and forms of myocardial infarction. High RC levels were associated with a higher incidence of young-onset AMI in patients with males, different LDL-c levels, and BMI levels (Supplementary Figure S1). Figure 2 also illustrates that the RC level was correlated with young-onset AMI in males (OR: 1.745, 95% CI: 1.408–2.161, *P* < 0.001), but not in females (OR: 0.884, 95% CI: 0.621–1.258, *P* = 0.493), after adjustment for other risk factors. Our findings indicate that irrespective of LDL levels being within the desired range, the presence or absence of hypertension, BMI, and the type of myocardial infarction, RC levels are significantly correlated with young-onset AMI (Figure 2; Supplementary Table S2).

## Discussion

This study suggested that RC were significantly associated with young-onset AMI in patients with T2DM, especially in males. We also demonstrated that RC is an independent risk factor for young-onset AMI, regardless of whether LDL-c levels are within the standard range.

In this study, we used two grouping methods to investigate the clinical characteristics of patients: tertiles and recommended values from a large perspective cohort study and obtained consistent conclusions. In T2DM patients, glucose and lipid metabolism disorders coexist and are positively correlated with BMI, which has been confirmed in previous studies. Elevated plasma triglycerides were once thought to be merely a consequence of obesity and insulin resistance in patients with T2DM, but later population-based large studies showed that hypertriglyceridemia can predict impaired glucose tolerance and T2DM, and is dose-dependent, independent of insulin resistance (Tricò et al., 2022; Bizzotto et al., 2021; Savage et al., 2007). Notably, although patients with high RC levels have a higher risk of young-onset AMI, there is still no difference in the severity of coronary artery lesions and cardiac function between different subgroups. Similar findings have been documented in prior research. Cordero et al. conducted a study on patients with acute coronary syndrome (ACS) and found that, in comparison to the low RC group, the high group exhibited a lower prevalence of prior strokes and heart failure, as well as reduced GRACE scores, but a higher proportion of premature ACS (Cordero et al., 2023). Possible reasons may be the following: 1) Individual differences in cholesterol metabolism, which reduces the deposition of cholesterol in the vessel wall and thus slows the progression of coronary artery disease; 2) It is related to the course of T2DM. In this study, patients with high RC levels were younger, had a higher proportion of STEMI, and stronger inflammatory response, suggesting that they may be related to acute unstable plaque rupture. Therefore, these patients have fewer complex coronary artery lesions and have a relatively better prognosis.

RC is an independent risk factor for young-onset AMI in T2DM patients. Previous studies have shown that RC significantly contributes to atherosclerotic plaque formation. A large primary prevention study found that elevated RC levels were associated with higher ASCVD risk, independent of traditional risk factors (Zhong et al., 2024). A study involving 8,523 hypertensive patients found a strong association between elevated RC levels and the presence of carotid plaques (Navarese et al., 2023). In old adults with hypertension, elevated RC was positively associated with carotid plaque, independent of LDL-c and other conventional risk factors (Tian et al., 2022). Another study nested within the Copenhagen General Population Study highlighted that elevated RC was associated with an increased risk of various atherosclerotic cardiovascular diseases, including myocardial infarction and ischemic stroke (Zhao et al., 2024). These studies provide robust evidence supporting the hypothesis that remnant cholesterol contributes to the development and progression of atherosclerotic plaques, highlighting its importance as a target for cardiovascular risk management. In mechanism, Large emulsified lipoprotein and VLDL particles are unable to traverse the endothelial barrier, but smaller remnants can not only penetrate the arterial intima but may be bound and retained by the connective tissue matrix. Each residual particle can carry up to 40 times more cholesterol than LDL-c, making arteriosclerosis more severe. Unlike LDL, residual particles do not require oxidation to be taken up by macrophages, leading to the formation of foam cells (Xiao et al., 2016; Salinas and Chapman, 2020; Schwartz and Reaven, 2012). Elevated levels of RC to elicit arterial wall inflammation and a multilevel cellular immune response (Bernelot Moens et al., 2017). Inflammatory responses and intimal lipid deposition lead to the formation of atherosclerotic plaques.

In our study, the risk of young-onset MI in the high RC group was higher than that in the low RC group, consistent with the findings from previous studies in other populations. Two prospective population cohort studies, The Copenhagen City Heart Study (CCHS) and the Copenhagen General Population Study (CGPS), both have been proved that elevated levels of calculated and directly measured RC was significantly associated with ASCVD. Meanwhile, the results of detecting the related gene variations that can raise remnant cholesterol in these cohorts show that specific genetic variations are causally related to high remnant cholesterol levels and the risk of acute cardiovascular disease (Varbo et al., 2013). Our study also demonstrated that the association between RC and young-onset MI is independent of LDL-c levels, meaning that RC can bring about remnant MI risk regardless of whether LDL-c levels have been achieved in patients with T2DM. This result suggests that for patients with T2DM, even after receiving statin therapy with an LDL-c ≤1.8 mmol/L, attention should still be paid to the residual cardiovascular risk associated with RC.

In addition, in subgroup analyses, we found sex differences between RC levels and early myocardial infarction. Lipid metabolism is significantly affected by estrogen and testosterone, and premenopausal women tend to have higher HDL and lower LDL due to estrogen secretion (Link and Reue, 2017). While estrogen levels drop during menopause, this increases LDL cholesterol and triglycerides and may increase the risk of cardiovascular disease. Higher testosterone levels in men can increase the risk of RC by lowering HDL and raising triglyceride levels. Triglyceride levels and RC can rise as a result of men’s eating habits, including eating more saturated fat and less fiber. There are also gender differences in physical activity, with men participating in all forms of sport. The distribution of body fat is also important; Men tend to have more visceral fat, which increases the risk of cardiovascular disease and RC (Piché et al., 2018; Gomez-Delgado et al., 2021; Muscella et al., 2020).

RC has emerged as a significant marker in cardiovascular research, particularly regarding its role in metabolic dysfunction and atherogenesis. RC as a marker of metabolic dysfunction. Elevated levels of RC are often indicative of underlying metabolic dysfunction, particularly disorders related to lipid metabolism (Heo and Jo, 2023). These metabolic disturbances can result in the overproduction of triglyceride-rich lipoproteins, leading to higher RC levels in circulation. Studies have shown that even when LDL-c is adequately managed, high RC levels can independently predict cardiovascular events, indicating that it reflects a broader spectrum of metabolic dysregulation beyond traditional lipid parameters (Quispe et al., 2021). Additionally, RC could directly contribute to atherogenesis through several mechanisms. High RC levels can activate endothelial cells, leading to oxidative stress and impaired endothelial function, which is a critical early step in atherosclerosis development (Sandesara et al., 2019). It can also promote inflammation and cytokine production, leading to plaque formation and progression. Research indicates that RC may be a better predictor of cardiovascular events than traditional lipid measures. However, it still has certain limitations. The composition of TRLs can vary widely among individuals due to factors like diet and metabolism. This variability means that the same RC value may not carry the same risk across different patients. Additionally, the calculation method does not directly measure remnant particles, instead, it estimates their presence based on other lipid components. But it is still an independent risk marker in clinical assessments.

Due to the limitations of RC detection methods, in clinical practice, on the basis of evaluating traditional risk factors, RC is used as an independent factor to assist physicians in assessing disease risk stratification of patients, especially for patients with pre-existing cardiovascular disease and poorly controlled LDL-c levels. Develop a protocol for regular monitoring of RC levels in patients known to be at cardiovascular risk or receiving lipid-lowering therapy. This ongoing evaluation can help tailor treatment adjustments and improve patient outcomes.

This study has some limitations. First, this was a single-center, retrospective and observational study. We included only the lipid levels of the patients at the time of admission, and there was a lack of lipid data before the onset of the disease, which could not fully represent the remnant cholesterol levels when the condition was stable. And our study lacked longitudinal or follow-up data to confirm causality. Further investigation with more follow-up data is needed in the future. Second, most patients with acute myocardial infarction have blood drawn via a vein upon admission, so the remnant cholesterol we calculate is a combination of fasting and non-fasting, to some extent, the remnant cholesterol level may be underestimated. Confounders of unknown variables such as medication adherence and dietary patterns that could lead to changes in RC levels were not corrected. Finally, due to a lack of information on the use of statins, we were unable to assess the impact of statins on RC, however, we evaluated lipid management indirectly based on LDL-c levels.

In conclusion, remnant cholesterol was a significant risk factor for young-onset AMI in patients with T2DM, independent of LDL-c level. More attention should be paid to the remnant cholesterol level in clinical work.

## Data Availability

The raw data supporting the conclusions of this article will be made available by the authors, without undue reservation.
