# Supplementary material for: The relationship between remnant cholesterol and young-onset myocardial infarction in patients with type 2 diabetes: a retrospective study
Source: Front Pharmacol. 2025 Mar 17;16:1512662. doi: 10.3389/fphar.2025.1512662 (PMC11955588; doi:10.3389/fphar.2025.1512662)
Supplement: Supplementary file 1 [file Table1.pdf]

Table S1 Vessels and clinical characteristics of study participants

Variables	T1(n=838) [a]	T2(n=843) [a]	T3(n=833) [a]	*P*	RC≤0.78mmol/L (n=1470) [b]	RC>0.78mmol/L (n=1044) [b]	*P*
Comorbidities, n (%)							
MI history	44(5.3)	47(5.6)	39(4.7) # †	0.705	78(4.6)	52(4.9)	0.717
Hypertension	516(61.6)	524(62.2)	536(64.3) # †	0.467	912(62.0)	664(63.6)	0.425
Stroke	118(14.1)	104(12.3)	77(9.2) # †	0.008	201(13.7)	98(9.4)	0.001
Cancer	19(2.3)	15(1.8)	16(1.9)	0.762	34(2.3)	16(1.5)	0.167
Pulmonary infection,	100(11.9)	102(12.1)	77(9.2) # †	0.113	176(11.9)	103(9.9)	0.097
Angiographic and procedural characteristics, n (%)							
Diseased vessels							
One	88(10.5)	88(10.4)	76(9.1) # †	0.063	155(10.5)	97(9.3)	0.074
Two	127(16.1)	140(17.6)	165(20.5) # †	0.063	236(16.0)	196(18.7	0.075
Three	575(72.8)	569(71.4)	560(69.7)	0.383	1000(68.0)	704(67.4)	0.749
PCI	697(83.2)	706(83.7)	735(88.2) # †	0.006	1229(83.6)	909(87.1)	0.016
PTCA	261(31.1)	234(27.8)	223(26.8) #	0.115	437(29.7)	281(26.9)	0.124
WBC (10^9/L)	8.36(6.56,10.59)	8.55(6.59,11.03)	9.34(7.42,11.66) # †	<0.001	8.37(6.56,10.65)	9.21(7.29,11.6)	<0.001
Hb (g/L)	136(122,148)	139(125,150)	143(130,155) # †	<0.001	137(123,149)	143(130,154)	<0.001
NT-proBNP (pg/ml)	927.9(300.8,2390.0)	895.75(292.43,2655.75)	587.2(187.03,1906.75) # †	0.13	928.2(301.5,2513)	643.8(200.55,2060)	0.039
CRP (mg/L)	10(10.00,23.38)	10(10.00,23.8)	10(10.00,15.55)	0.013	10(10.00,24.65)	10(10.00,15.75)	0.005
hs-TnT (ng/ml)	0.34(0.09,1.17)	0.41(0.1,1.38)	0.35(0.08,1.18)	0.776	0.38(0.09,1.23)	0.37(0.08,1.24)	0.482
CKMB (U/L)	24(14,74)	25(15,67)	27(15,77) # †	0.118	25(14,72)	27(15,77)	0.523
eGFR (MDRD) (ml/min/1.73m^2)	101.76(77.09,125.2)	102.59(75.23,129.39)	103.35(79.99,128.03)	0.21	101.83(76.73,126.65)	103.76(78.7,128.4)	0.104
BUN (mmol/L)	6(4.71,7.59)	5.96(4.76,7.56)	6.06(4.96,7.6)	0.926	6(4.72,7.59)	6.02(4.89,7.57)	0.584
CRE (umol/L)	65(53,82)	65(52,80)	65(53,81)	0.645	65(53,81)	65(53,81)	0.544
ALB (g/L)	36.1(32.9,38.9)	37.3(34.5,40.0)	40.1(36.9,43.3) †	<0.001	36.4(33.5,39.4)	39.6(36.4,42.8)	<0.001
AST (U/L)	36(22,82)	38(24,84.5)	42(25,87) †	0.505	37(23,82)	42(25,88)	0.559
ALT (U/L)	30(20,45)	31(20,47)	30(21,47)	0.845	30(20,46)	30(21,46)	0.771

Continuous variables are shown as M±SD or quartiles, and classification variables are shown as percentages. [a]: The subjects were divided into three groups according to remnant cholesterol tertiles. [b]: The subjects were divided into two groups according to the PREDIMED cohort study. Chi-square test, Kruskal-Wallis H test were used for statistical analysis. [#]: Compared with T1 group, there was a statistical difference. [†]: Compared with T2 group, there was a statistical difference. PCI: Percutaneous coronary intervention; PTCA: percutaneous transluminal coronary angioplasty; Hb: Hemoglobin; WBC: White blood cell; NT-proBNP: N-terminal B-type pro-brain natriuretic peptide; CRP: C-reactive protein; Hs-TnT: High-sensitivity troponin T;

CKMB: Creatine kinase isoenzyme; GFR: Glomerular filtration rate; BUN: Blood urea nitrogen; CRE: Creatinine; ALB: Albumin; AST: Aspartate aminotransferase; ALT: Alanine aminotransferase.
