# Supplementary material for: The relationship between remnant cholesterol and young-onset myocardial infarction in patients with type 2 diabetes: a retrospective study
Source: Front Pharmacol. 2025 Mar 17;16:1512662. doi: 10.3389/fphar.2025.1512662 (PMC11955588; doi:10.3389/fphar.2025.1512662)
Supplement: Supplementary file 2 [file Table2.pdf]

Table S2 Subgroups analysis of RC and young-onset AMI

Variables	OR (95% CI)	*P*
**LDL-c<1.8**		
RC≤0.78	0.749 (0.491, 1.142)	0.179
RC>0.78	1.336 (0.876, 2.037)	
**LDL-c≥1.8**		
RC≤0.78	0.713 (0.572, 0.888)	**0.003**
RC>0.78	1.403 (1.126, 1.749)	
**Male**		
RC≤0.78	0.605 (0.486, 0.755)	**<0.001**
RC>0.78	1.625 (1.325, 2.060)	
**Female**		
RC≤0.78	1.242 (0.809, 1.907)	0.322
RC>0.78	0.805 (0.524, 1.237)	
**NSTEMI**		
RC≤0.78	0.656 (0.487, 0.885)	**0.006**
RC>0.78	1.524 (1.130, 2.054)	
**STEMI**		
RC≤0.78	0.739 (0.573, 0.954)	**0.02**
RC>0.78	1.352 (1.048, 1.746)	
**HBP**		
RC≤0.78	0.758 (0.589, 0.975)	**0.031**
RC>0.78	1.320 (1.026, 1.698)	
**non-HBP**		
RC≤0.78	0.618 (0.456, 0.837)	**0.002**
RC>0.78	1.618 (1.194, 2.192)	
**BMI≤24**		
RC≤0.78	0.665 (0.489, 0.904)	0.009
RC>0.78	1.505 (1.106, 2.046)	
**BMI>24**		
RC≤0.78	0.702 (0.550, 0.895)	0.004
RC>0.78	1.425 (1.117, 1.818)	

The model was adjusted for gender, SBP, DBP, BMI, MI history, HAblc, CKMB, eGFR. $P<0.05$ indicated that the difference was statistically significant. In each layer, all models are not adjusted for the variables themselves. OR: odds ratios; CI: confidence interval. LDL-c: Low-density lipoprotein cholesterol; STEMI: ST-elevation myocardial infarction; NSTEMI: Non-ST-elevation myocardial infarction; HBP: hypertension; BMI: body mass index.
